# A PROACTIVE CARE PROGRAM FOR FRAIL OLDER PEOPLE LIVING AT HOME: A 2-YEAR EVALUATION

**DOI:** 10.1093/geroni/igz038.178

**Published:** 2019-11-08

**Authors:** Nienke Bleijenberg, Jonna Rijksen, Marieke J Schuurmans, Niek J de Wit

**Affiliations:** 1 University of Applied Sciences Utrecht, Utrecht, Netherlands; 2 Julius Center for Health Sciences and Primary Care, University Medical Center Utrecht, Utrecht, Utrecht, Netherlands; 3 Education Center, UMC Utrecht Academy, University Medical Center Utrecht, Utrecht, Utrecht, Netherlands; 4 Julius Center for Health Sciences and Primary Care, University Medical Center Utrecht, Utrecht, Overijssel, Netherlands

## Abstract

This study presents the long-term evaluation and impact of a proactive primary care program that aims to reduce acute healthcare consumption and preservation of daily functioning of frail older people living at home. For nine years the program has been adapted and evaluated. We present the results of our implementation study with two-year follow-up. 53 general practices (GP) participated. They provided care to approximately 35000 people aged 65 years and older. Data was extracted from routine primary care data, hospital data and social care data from the municipality. Important outcomes were: number of GP visits, house visits by GP, out-of-hours primary care visits, emergency room (ER) visits, hospital admission, social support, self-sufficiency and wellbeing. After implementing the program, a significant reduction in acute visits (ER and out-of-hour visits) was observed. GP contacts and visits were also significantly increased. The program was well perceived by professionals.

